# A Qualitative Study of Healthcare and Aging While Incarcerated

**DOI:** 10.1093/geroni/igaf122.092

**Published:** 2025-12-31

**Authors:** Michelle Suh, Megan Benavides, Stephen Chen, Annika Bhananker, Sumin Yoon, Marc Robinson, Josiah Rich, Anita Chary

**Affiliations:** Brown University, Providence, Rhode Island, United States; Baylor College of Medicine, Houston, Texas, United States; Baylor College of Medicine, Houston, Texas, United States; Rice University, Houston, Texas, United States; Houston, Texas, United States; Baylor College of Medicine, Houston, Texas, United States; Brown University, Providence, Rhode Island, United States; Baylor College of Medicine, Houston, Texas, United States

## Abstract

Older adults comprise the fastest growing demographic in correctional facilities in the United States. While incarcerated, prisoners not only develop chronic conditions but also experience accelerated aging. However, little is known about how older adults navigate the correctional environment, aging, and healthcare. We sought to explore experiences of aging and healthcare in incarcerated older adults. We conducted semi-structured qualitative interviews with 16 incarcerated older adults (>50 years old) from the county jail who were receiving care in a public hospital emergency department. Interviews focused on experiences of aging and healthcare while incarcerated. We used an inductive approach to code data and identify overarching themes. Two major themes emerged. First, participants reported difficulty navigating the jail environment due to aging and disabilities. Examples included using the top bunk bed due to social pressure from other inmates, difficulty reading printed instructions due to visual impairments, and a Parkinson’s tremor impeding the grasp of eating utensils. Participants relied on other inmates for assistance but were vulnerable to exploitation, such as commissary fund theft. Second, participants faced numerous challenges attempting to access healthcare. Issues included confusion regarding the protocol for accessing care especially digital kiosks, interruptions in care for chronic conditions including lapses in daily medications, and correctional staff skepticism about medical concerns. Despite a constitutional right to healthcare and high rates of medical comorbidities, older prisoners experienced a challenging physical environment and difficulty accessing healthcare. Further work is needed to identify potential age-friendly modifications to the correctional environment and improved healthcare delivery.

